# Immunohistochemical Evidence of Telocytic Stroma Associated with Tumor Grade and Acinar Heterogeneity in Prostate Cancer

**DOI:** 10.3390/ijms27031537

**Published:** 2026-02-04

**Authors:** Eduardo P. Júnior, Mário F. R. Lima, Lúcia P. F. Castro, Pablo V. N. Ramos, Juan C. M. Onofre, Rafaela S. Souza, Vivian Resende, Clémence Belleannée, Gabriel Campolina-Silva, Marcelo Mamede

**Affiliations:** 1 Departamento de Anatomia Patológica e Medicina Legal, Faculdade de Medicina, Universidade Federal de Minas Gerais, Avenida Prof. Alfredo Balena, 190, Belo Horizonte 30130-100, MG, Brazil; 2 Laboratório Analys Patologia, Rua Pouso Alegre, 110, Floresta, Belo Horizonte 31110-010, MG, Brazil; 3 Departamento de Cirurgia, Faculdade de Medicina, Universidade Federal de Minas Gerais, Belo Horizonte 30130-100, MG, Brazil; 4 CHU de Quebec Research Center, Université Laval, Quebec, QC G1V 0A6, Canada; 5 Departamento de Anatomia e Imagem, Faculdade de Medicina, Universidade Federal de Minas Gerais, Belo Horizonte 30130-100, MG, Brazil

**Keywords:** prostate cancer, reactive stroma, telocytes, myofibroblasts, immunohistochemistry, tumor microenvironment

## Abstract

Prostate cancer (PCa) progression involves dynamic interactions between neoplastic cells and the reactive stroma (RS). Although myofibroblasts are established components of the RS, the role of other stromal populations, such as telocytes, remains poorly understood. This study investigated the presence and distribution of a telocytic stromal phenotype (CD34^+^/Vimentin^+^) in PCa across different histological grades and acinar patterns. We used digital image analysis and standardized immunohistochemistry to assess biopsy samples from 120 patients with confirmed PCa. The telocytic phenotype showed a heterogeneous distribution and was significantly enriched in high-grade tumors and specific acinar architectures, particularly Patterns B and D. In contrast, well-differentiated regions exhibited lower telocyte density, resembling non-neoplastic prostate tissue. Although the myofibroblastic phenotype (α-SMA^+^/Vimentin^+^/CD34^−^) also increased overall with tumor grade and varied across acinar patterns, this association was comparatively weaker and less statistically robust than that observed for telocytes. These results suggest that stromal remodeling encompasses a spectrum of cellular phenotypes influenced by local architectural constraints. It is proposed that telocytes serve as key mediators of tissue organization and biomechanical signaling, contributing to a feedback loop that promotes tumor progression. Combining acinar architecture with stromal phenotyping provides a refined framework for understanding epithelial–stromal co-evolution in PCa.

## 1. Introduction

Prostate cancer (PCa) is one of the most prevalent malignancies among men worldwide [1,2]. While most attention has focused on understanding genetic and molecular alterations within neoplastic epithelial cells, increasing evidence underscores the critical role of the tumor microenvironment in cancer progression [3], particularly the stromal compartment. The concept of reactive stroma (RS) in cancer parallels wound healing, wherein stromal cells respond dynamically to epithelial transformation [4,5,6,7]. In prostate tumors, RS has been associated with disease progression, tumor aggressiveness, and therapeutic outcomes, highlighting the functional interplay between stromal and epithelial compartments. RS in PCa typically features an enrichment of myofibroblasts, marked by strong immunopositivity for α-smooth muscle actin (α-SMA) and Vimentin, with variable expression of terminal muscle differentiation markers such as Desmin or Calponin [4,8,9]. Recent studies, however, have expanded our understanding of the cellular composition of the RS as well as its influence on tumor biology, highlighting the contributions of several other connective tissue cell types that collectively shape tumor-stroma interactions [10,11].

Telocytes are interstitial cells present in many organs, characterized by their long cytoplasmic projections (telopodes) and concomitant expression of CD34 and Vimentin [12]. In the prostate gland, longitudinal studies in rodents have demonstrated that telocytes are an integral component of the stroma during prostate development and adulthood [13,14]. Their abundance increases in the aging prostate, where they are likely to contribute to extracellular matrix remodeling and epithelial growth, partly through secretion of TGF-β [14]. In humans, telocytes have also been reported in higher numbers within benign periurethral stromal nodules and tumor-associated RS [15], suggesting a role for these cells in prostate tumorigenesis [16]. Nevertheless, the prevalence, distribution, and biological relevance of telocytic stromal phenotypes in human PCa remain poorly defined.

To investigate the contribution of RS heterogeneity to PCa biology, we evaluated the distribution of myofibroblastic and telocytic stromal phenotypes in human prostate cancer biopsies using immunohistochemistry for well-established stromal markers in relation to tumor grade and acinar architecture.

## 2. Results

### 2.1. Patient Cohort Characteristics

The study cohort consisted of 120 patients with treatment-naïve prostate cancer, distributed evenly across Gleason score groups: GS 3 + 3 (Group 1, *n* = 30), GS 3 + 4 (Group 2, *n* = 30), GS 4 + 3 (Group 3, *n* = 30), and GS ≥ 8 (Group 4, *n* = 30). The median patient age was comparable across groups (median = 66 years; Q1–Q3 range = 61–71 years). The overall median serum prostate-specific antigen (PSA) level was 7.5 ng/mL (interquartile range [IQR] = 11.7 ng/mL). As expected, patients with high-grade disease (Group 4) exhibited higher PSA levels (median = 11.48 ng/mL; IQR = 39.88 ng/mL) compared with those in Groups 1–3, in whom median PSA values ranged from 6.65 to 8.88 ng/mL.

Of 327 regions of interest (ROIs; 1–3 per case) initially identified within diagnostic biopsy cores based on reactive peritumoral stroma features [17], 260 ROIs were retained after excluding areas with tissue loss or absent tumor. Serial sections of these cores enabled consecutive immunohistochemistry (IHC) staining for smooth muscle–associated markers (α-SMA, Desmin) and telocyte-enriched markers (CD34, Vimentin). The retained ROIs, averaging two per case, were evenly distributed across Gleason score Groups (n = 61, 65, 67, and 67 for Groups 1–4), providing spatially representative sampling of the peritumoral stroma and capturing histopathological heterogeneity while minimizing sampling bias.

### 2.2. Distribution of α-SMA, Desmin, Vimentin, and CD34 in Normal and PCa Stroma

**Figure 1:**
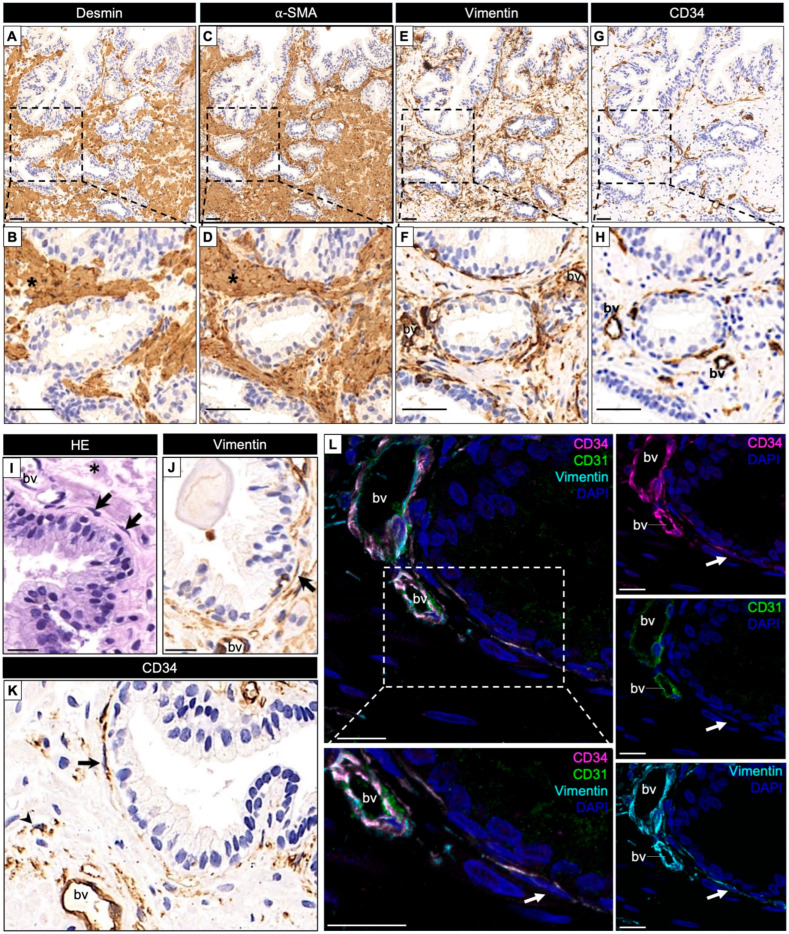
Immunohistochemical characterization of stromal cell populations in non-neoplastic human prostate. (**A**–**D**) Desmin and α-SMA immunostaining showing well-organized smooth muscle bundles (*) and periacinar myoid cells in histologically normal prostatic regions (higher magnification in (**B**,**D**)). (**E**,**F**) Vimentin expression in periacinar stromal cells and scattered interstitial cells, including blood vessels (bv), reflecting the heterogeneous distribution of this marker in the prostatic stroma. (**G**,**H**) Overview of CD34 immunoreactivity in non-neoplastic prostatic stroma, predominantly associated with blood vessels (bv) and scattered periacinar stromal cells. (**I**–**K**) Histological and immunohistochemical sections showing fusiform stromal cells with elongated nuclei and slender cytoplasmic processes (black arrows), consistent with telocyte-like morphology, as observed in hematoxylin–eosin (HE) staining (**I**), Vimentin immunoreactivity (**J**), and CD34 immunostaining (**K**). (**L**) Multiplex immunofluorescence showing highlighted periacinar CD34^+^ stromal cells (white arrows) co-expressing CD34 (magenta) and Vimentin (cyan), while lacking expression of the endothelial marker CD31 (green), supporting their non-endothelial stromal identity and consistent with telocyte-like cells. Nuclei are counterstained with DAPI (blue). Scale bars = 20 µm.

We first assessed staining in histologically normal regions present within biopsy cores. These regions, although not included among the ROIs used for quantitative analysis, provided an important baseline of marker localization in intact prostatic architecture. Both Desmin and α-SMA showed strong and diffuse labeling, consistent with well-preserved smooth muscle bundles (Figure 1A–D). Vimentin exhibited a heterogeneous distribution, labeling periacinar stromal cells and scattered interstitial elements (Figure 1E–J). CD34 immunoreactivity was primarily observed in vascular endothelial cells; however, in addition to vascular profiles, scattered CD34^+^ stromal cells with elongated cytoplasmic processes were detected in the periacinar stroma (Figure 1G–K). These cells exhibited morphological features consistent with telocytes, being located either adjacent to the epithelial basement membrane or dispersed throughout the stroma (Figure 1K,L). Co-labeling with Vimentin and the absence of CD31 expression supported their stromal, non-endothelial identity (Figure 1L). Although CD34 is a canonical marker of hematopoietic stem and progenitor cells, it is also expressed by certain non-hematopoietic stromal populations, including telocytes [12,14]. These observations provide qualitative confirmation that CD34^+^/Vimentin^+^ cells located outside blood vessels correspond to telocytes.

**Figure 2:**
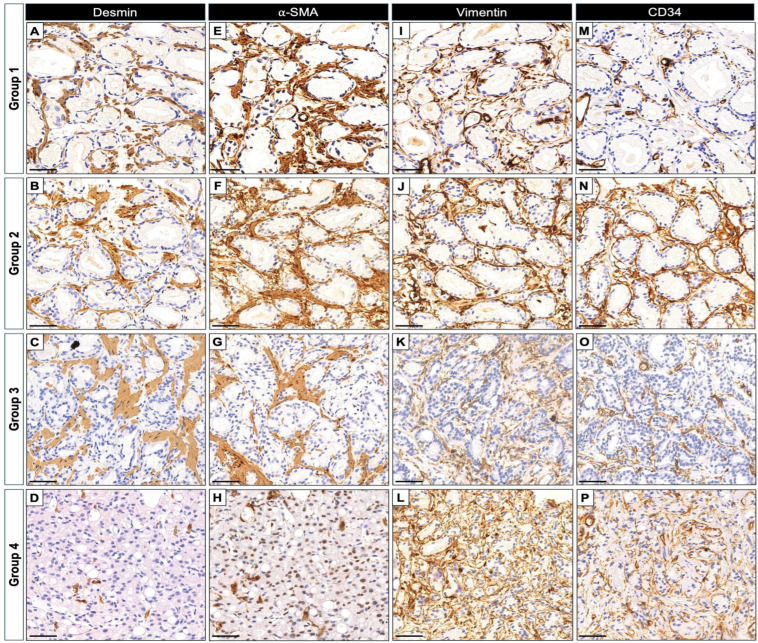
Representative immunohistochemical patterns of Desmin, α-SMA, Vimentin, and CD34 expression across Gleason groups in human prostate tumors. (**A**–**D**) Desmin staining showing a consistent and progressive reduction from low- to high-grade tumors, with intertumoral variability, and residual immunoreactivity mainly restricted to stromal smooth muscle fibers in Group 4. (**E**–**H**) α-SMA displays a commonly observed moderate increase in immunoreactivity in Groups 1–3, followed by a decrease in Group 4, where labeling is predominantly confined to residual stromal smooth muscle fibers, partially overlapping with the Desmin pattern. (**I**–**L**) Vimentin and (**M**–**P**) CD34 exhibit a commonly observed progressive increase in both staining intensity and spatial distribution with tumor progression, reflecting enhanced stromal reactivity and overlapping expression patterns. Gleason groups were defined as follows: Group 1, Gleason score 3 + 3; Group 2, 3 + 4; Group 3, 4 + 3; Group 4, Gleason score ≥ 8. Scale bars = 50 µm.

Next, we focused our analysis on the tumor-associated stroma. Qualitative assessments pointed to substantial heterogeneity in marker distribution across Gleason groups (Figure 2). In low-grade tumors, CD34 expression largely mirrored that of non-neoplastic tissue, being confined to vascular structures and sparse periacinar cells. However, in high-grade tumors, CD34^+^ fusiform cells became increasingly prominent within the stromal compartment, reaching a peak in Group 4 (GS ≥ 8) (Figure 2). Vimentin expression showed a trend toward higher levels in advanced tumors and exhibited a distribution pattern partially overlapping with CD34^+^ stromal cells (Figure 2). ROIs with apparently high levels of CD34 and Vimentin often showed a slight reduction in α-SMA and Desmin expression, particularly in high-grade lesions (Figure 2).

### 2.3. Quantitative Association of Stromal Marker Expression with Gleason Grade in PCa

**Figure 3:**
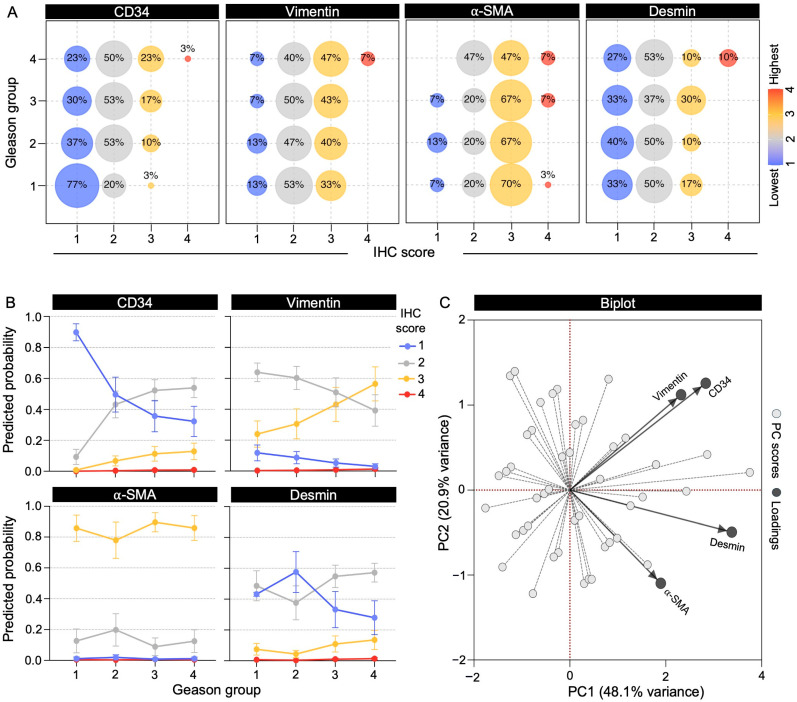
Stromal marker immunoexpression according to Gleason grade groups. (**A**) Bubble plots showing the percentage of patients exhibiting each IHC score per Gleason group. (**B**) CLMM-derived predicted probabilities ± CI 95% of marker expression across Gleason grades. (**C**) Principal component analysis (PCA) biplot integrating stromal marker expression across patients. Vectors represent marker loadings on the first two principal components, summarizing overall variance structure and relationships among markers.

To quantitatively assess stromal marker expression across ROIs, pixel-level intensities for CD34, Vimentin, α-SMA, and Desmin were measured and scored into four ordinal categories (1 = negative, 2 = low, 3 = moderate, 4 = high), based on the dominant percentage of pixels per category. The distribution of marker-specific IHC scores presented among patients showed that CD34 and Vimentin tended to shift toward higher expression scores from Gleason group 1 to groups 2–4, whereas α-SMA and Desmin remained largely stable (Figure 3A). Assessments using cumulative link mixed models (CLMM), including patient as a random effect, revealed that CD34 demonstrated a strong positive linear association with tumor grade, corresponding to an eight-fold increase in the odds of observing a higher expression score for each stepwise increase in Gleason group (OR = 8.09, 95% CI 2.81–23.27, *p* = 0.0001) (Table 1). Vimentin also showed a significant positive association, although with a more moderate effect size (OR = 3.00, 95% CI 1.20–7.50, *p* = 0.019; Table 1). In contrast, neither α-SMA nor Desmin demonstrated significant associations with tumor grade, which may be partly explained by the higher inter-patient variability observed for these markers (variance of random intercept: SMA = 8.22, Desmin = 5.37) compared to CD34 (3.31) and Vimentin (3.73), potentially masking smaller effects (Table 1). Age and PSA were not significantly associated with expression in any model (Supplementary Table S1).

Predicted probabilities derived from the CLMM further illustrated these trends (Figure 3B), with a marked decrease in low CD34 expression from Gleason group 1 to group 4, paralleled by a progressive enrichment of intermediate and high expression categories. Vimentin exhibited a similar but less pronounced pattern, whereas α-SMA and Desmin remained comparatively stable across grades (Figure 3B). Together, these findings indicate that CD34, and to a lesser extent Vimentin, undergo grade-dependent modulation in PCa, supporting the presence of dynamic stromal remodeling associated with tumor progression.

### 2.4. Telocytic Stromal Phenotype Is Enriched in Advanced Prostate Cancer

We evaluated the combinatorial expression of the immunohistochemical markers analyzed in this study to better understand stromal heterogeneity. Principal component analysis (PCA) supported coordinated variation among these markers, showing that, although all four align along the principal component 1 (PC1), CD34 and Vimentin clustered together and were clearly separated from α-SMA and Desmin along PC2 (Figure 3C). These patterns indicate that our immunohistochemistry approach is suitable for the identification of two distinct stromal phenotypes within the tumor microenvironment. The telocytic phenotype was defined by high CD34 and Vimentin expression (scores 3–4), whereas the myofibroblastic phenotype was characterized by high α-SMA and Vimentin expression combined with low CD34 (scores 1–2).

As summarized in Table 2, the telocytic phenotype was identified in 16.2% of all ROIs (42/260) and in 25% of patients (30/120) overall. Although relatively low in occurrence, the telocytic phenotype prevalence increased progressively with tumor grade, rising from 6.6% in Group 1 to 22.4% in Group 4 at the ROI level, and from 10.0% to 33.3% at the patient level (Table 2). Bayesian logistic regression models incorporating patient as a random intercept supported these observations. Moving from Group 1 to higher Gleason groups was associated with substantially increased odds of detecting the telocytic phenotype (linear trend OR = 3.08, 95% CI 1.31–6.25; Table 2). For instance, compared with Group 1, the odds roughly doubled in Group 2 and were about four times higher in Groups 3 and 4 (Table 2), illustrating a clear, grade-dependent increase in telocytic enrichment.

In contrast, the myofibroblastic phenotype was more frequently detected, present in approximately 32% (83/260) of ROIs and 42% (50/120) of patients overall. Although its prevalence increased from lower to higher Gleason grade groups, this association was weaker and characterized by greater uncertainty compared with the telocytic phenotype (OR = 1.86, 95% CI 0.92–3.36) (Table 2). As in the telocytic model, patient age and PSA levels showed no relevant contribution to the likelihood of phenotype detection (Supplementary Table S2).

These findings indicate that telocytic enrichment reflects a more dynamic, grade-dependent stromal response than the myofibroblastic phenotype, highlighting that distinct stromal phenotypes contribute differently to tumor aggressiveness.

### 2.5. Differential Incidence of Telocytic and Myofibroblastic Stromal Phenotypes According to Acinar Architecture in Prostate Cancer

In addition to tumor grade based on Gleason score, we evaluated the distribution of telocytic and myofibroblastic phenotypes in relation to four distinct acinar arrangements identified in the analyzed samples, which are: Pattern A, comprising well-developed glands with lumina, including both larger acini and microacini > 50 µm in diameter; Pattern B, consisting primarily of regular peripheral microacini with a maximum diameter of 50 µm; Pattern C, characterized by cribriform or multiluminal epithelial sheets; and Pattern D, represented by solid epithelial strands, nests, or isolated infiltrating cells lacking luminal differentiation [17]. These patterns not only capture the progressive disruption of glandular architecture that underlies the Gleason grading system but have also been associated with features of reactive stroma [17].

**Figure 4:**
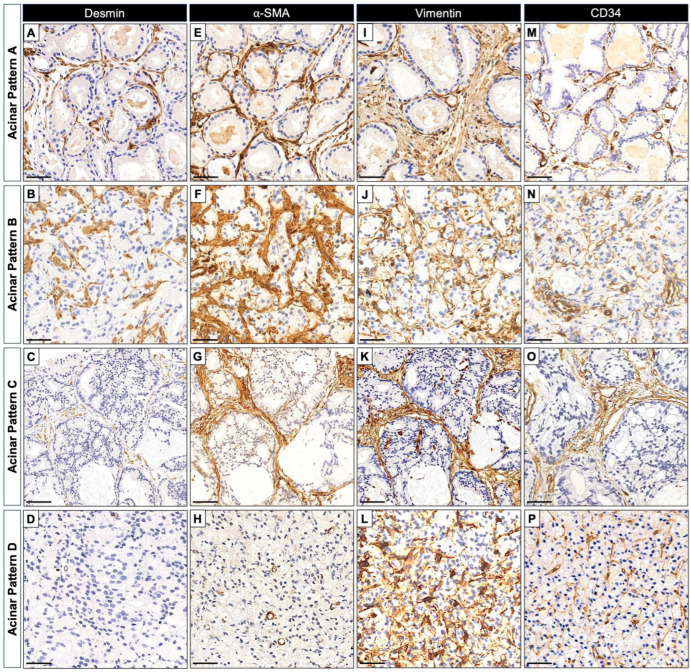
Representative immunohistochemical images illustrating the predominant expression and distribution patterns of stromal markers across acinar patterns in human prostate tumors. (**A**–**D**) Desmin immunoreactivity shows a frequently observed progressive reduction across acinar patterns, with a marked decrease in Pattern D. (**E**–**H**) α-SMA displays a commonly observed moderate increase in immunoreactivity in Patterns A–C, followed by a reduction in Pattern D, partially overlapping with the Desmin pattern. (**I**–**L**) Vimentin and (**M**–**P**) CD34 exhibit a frequently observed progressive increase in both staining intensity and spatial distribution across acinar patterns, particularly in Pattern D. Scale bars = 50 µm.

A total of 245 ROIs with clearly defined acinar patterns (APs) were included in the analysis, while 15 ROIs lacking a distinct acinar profile were excluded. At the ROI level, the telocytic phenotype was present in 23.3% (57/245) of regions, with a marked variation across APs: 7.4% in Pattern A, 34.4% in Pattern B, 10.5% in Pattern C, and 42.9% in Pattern D (Table 3). Patient-level analyses mirrored these trends, with telocytic enrichment observed in 12.8%, 44.2%, 14.3%, and 60.0% of patients across Patterns A to D, respectively (Table 3). Similarly, the myofibroblastic phenotype was detected in 30.6% (75/245) of ROIs overall, ranging from 21.1% in Pattern A to 41.7% in Pattern B, 15.8% in Pattern C, and 34.3% in Pattern D. At the patient level, corresponding frequencies were 23.4%, 44.2%, 21.4%, and 53.3%, respectively (Table 3). These distributions suggest that both phenotypes are most likely associated with acini architectures. Representative images for each individual marker in relation to APs are shown in Figure 4.

**Figure 5:**
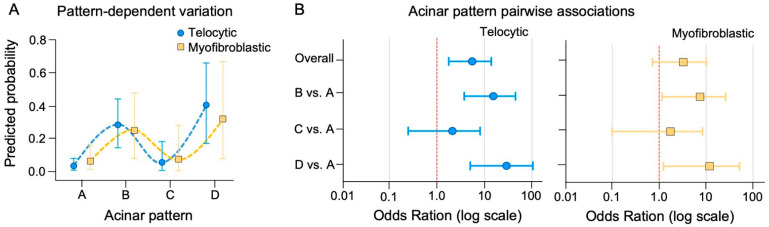
Pattern-dependent variation and pairwise associations of stromal phenotypes across acinar architectures. (**A**) Posterior predicted probabilities of stromal phenotype presence across APs (A–D), illustrating pattern-dependent and non-linear variation along the architectural continuum from well-formed glands to poorly differentiated structures. Vertical bars represent 95% CI. (**B**) Forest plots showing pairwise associations between APs expressed as odds ratios (ORs) with 95% CI on a logarithmic scale. ORs were derived from the linear polynomial contrast of the Bayesian multilevel logistic regression model (Table 3) and expressed as comparisons of Patterns B–D relative to Pattern A. The vertical dashed red line denotes the null value (OR = 1).

Bayesian multilevel logistic regression confirmed these observations. A positive linear trend was observed across AP (A–D), indicating that, on average, higher patterns were associated with increased odds of telocytic enrichment (linear trend OR = 5.55, 95% CI 1.78–13.97). The association also included a strong cubic component (OR = 10.60, 95% CI 2.50–33.04), reflecting non-linear behavior with intermediate attenuation at Pattern C. Pairwise comparisons using the full linear predictor revealed markedly higher odds of telocytic enrichment in Patterns B (OR = 15.39, 95% CI 3.77–45.10) and D (OR = 29.20, 95% CI 5.04–104.33) relative to Pattern A, whereas Pattern C showed attenuated enrichment (OR = 2.13, 95% CI 0.25–8.15) (Figure 5).

In contrast, the myofibroblastic phenotype exhibited a more modest and less consistent association with acinar architecture. A positive linear trend was observed across AP (linear trend OR = 3.28, 95% CI 0.72–10.15), accompanied by a cubic component (OR = 6.86, 95% CI 1.11–24.34) reflecting intermediate attenuation in Pattern C. Pairwise comparisons using the full linear predictor revealed higher odds of myofibroblastic enrichment in Patterns B (OR = 7.48, 95% CI 1.16–26.00) and D (OR = 11.91, 95% CI 1.23–51.76) relative to Pattern A, whereas Pattern C showed attenuated enrichment (OR = 1.75, 95% CI 0.10–8.44). This pattern is largely explained by the low number of events in Pattern C and greater variability across patients, which contributed to wider confidence intervals and reduced global effect sizes (Table 3, Figure 5).

Together, our findings indicate that both telocytic and myofibroblastic phenotypes were enriched in AP B and D, with stronger and more consistent associations for the telocytic phenotype. Notably, these results were independent of patient age and PSA levels (Supplementary Table S3), highlighting the differential sensitivity of stromal phenotypes to acinar architecture.

## 3. Discussion

Reactive stroma (RS) has long been recognized as a biologically active component of prostate cancer (PCa), classically defined by a shift from a differentiated, smooth-muscle-rich stroma to an activated, fibroblast-dominated phenotype [4,6,18]. Early landmark studies demonstrated that stromal activation, characterized by loss of smooth muscle differentiation, extracellular matrix (ECM) remodeling, and increased fibroblastic features, is closely associated with tumor progression and adverse clinical behavior, including biochemical recurrence [4,19]. In this context, RS has been viewed as a relatively uniform response accompanying epithelial malignancy.

The present study expands this classical view by demonstrating that stromal remodeling in PCa is not uniform, but reflects distinct biological states associated not only with tumor grade (as determined by Gleason score), but also and perhaps more critically, with specific AP. By integrating immunohistochemical profiling with detailed acinar pattern analysis, we show that a telocytic stromal phenotype—defined by combined high expression of CD34 and Vimentin—is progressively enriched in high-grade tumors and specific neoplastic acinar architectures. In contrast, the myofibroblastic phenotype exhibits a more modest and less consistent association with tumor progression. These findings support the concept that stromal heterogeneity in PCa extends beyond a myofibroblast-dominated model and suggest that non-classical stromal cell states contribute to RS organization in a manner tightly linked to epithelial and acinar architectural features.

Immunohistochemical profiling has been key to characterizing the prostatic stromal compartment, as the markers commonly used reflect cell populations with distinct roles in stromal remodeling and tumor progression [4]. For instance, increased Vimentin expression and reduced levels of smooth muscle markers, such as Desmin, have been associated with aggressive PCa and poorer clinical outcomes [4,8]. However, the interpretation of these changes has evolved with the recognition that stromal cell composition is more complex than initially appreciated. There is ongoing debate over whether cancer-associated fibroblasts (CAFs) and myofibroblasts are distinct stromal cell types with separate origins or represent different activation states within a single stromal lineage continuum [20,21].

Recent single-cell RNA sequencing (scRNA-seq) studies have reinforced this concept by identifying CAFs as one of the most abundant and functionally diverse components of the tumor microenvironment (TME) [22]. In PCa, CAF heterogeneity has been broadly categorized into myofibroblast-like CAFs (myCAFs), inflammatory CAFs, and adipogenic CAFs [22,23,24]. MyCAFs are consistently characterized by high expression of contractile and cytoskeletal markers, including ACTA2 (α-SMA) and VIM (Vimentin), as well as by ECM-related transcriptional programs. In this context, our identification of myofibroblast-enriched ROIs based on strong α-SMA and Vimentin immunoreactivity is conceptually aligned with myCAF states described in single-cell studies. Nevertheless, in our cohort, the odds of detecting a myofibroblastic phenotype showed only a weak upward trend from low- to high-grade tumors, with wide credible intervals overlapping the null value, indicating substantial inter-patient variability. This aligns with studies suggesting that myCAF enrichment alone may not fully capture the complexity of stromal dynamics associated with PCa aggressiveness [23,24].

Accumulating evidence indicates that the RS compartment in PCa is highly heterogeneous and encompasses mesenchymal cell states that extend beyond classical fibroblasts, smooth muscle cells, and CAFs [10,11,12,24]. Ultrastructural, immunophenotypic, and spatial studies in both human and rodent prostate have identified telocytes as integral components of the prostatic stroma [13,14,15,16]. Formally named by Popescu and Faussone-Pellegrini in 2010 [12], telocytes are stromal cells featuring extremely long and thin cytoplasmic extensions, termed telopodes. These structures are thought to facilitate long-range stromal–epithelial communication and contribute to tissue compartmentalization and architectural organization [12,13,14,15]. In our study, telocyte-enriched regions were identified using combined CD34 and Vimentin expression. While these markers are not entirely specific to telocytes, their combined use within a spatiotemporal context has been shown to reliably distinguish telocytes from other stromal cell types, including in the prostate gland [13,14,15,16]. Notably, prostatic telocytes have not yet been resolved as discrete clusters in single-cell transcriptomic datasets [10,11,25], likely due to limitations imposed by dissociation-based approaches. These factors may include loss of spatial context, telopode fragility, and low RNA content. In this setting, immunohistochemistry remains particularly valuable, as it allows assessment of telocyte presence while preserving tissue architecture and morphology.

Consistent with this view, our spatially resolved analyses identify a CD34^+^/Vimentin^+^ stromal population with telocytic morphology that becomes progressively enriched in advanced PCa. Although telocytic profiles accounted for a smaller fraction of ROIs overall, their frequency increased markedly with Gleason grade, with the odds of detecting a telocytic enrichment roughly doubling in intermediate-grade tumors and increasing about fourfold in high-grade tumors compared with low-grade lesions. This enrichment showed a stronger, more consistent association with a shift toward poorer histological differentiation than did the myofibroblastic phenotype, suggesting that telocytic accumulation represents a dynamic stromal response accompanying disease advancement rather than a static background feature of the prostate stroma.

Importantly, telocytic enrichment was not uniformly distributed across tumor regions but was strongly associated with specific acinar architectures. Telocytes were markedly enriched in poorly differentiated patterns, particularly Patterns B and D, while well-differentiated acinar structures (Pattern A), resembling non-neoplastic tissue, exhibited low telocyte density. These observations support the notion that epithelial–stromal interactions are highly context-dependent and shaped by local architectural constraints. Our previous work linked Pattern B architecture to increased collagen deposition and acinar disorganization [17], and the present findings suggest that this specific microenvironment may favor telocyte recruitment or expansion.

From a broader perspective, these data support a model in which acinar architecture acts as a spatial scaffold, guiding stromal cell recruitment and specialization. Telocytes, in particular, may contribute to stromal compartmentalization and neoplastic rearrangements by modulating extracellular matrix organization, collagen remodeling, and tissue biomechanics [12,13,14,15,26,27,28,29,30,31]. They form extensive intercellular networks and interact with fibroblasts, smooth muscle cells, vascular elements, and epithelial structures, positioning them as potential mediators of both biomechanical and biochemical signaling within the tumor microenvironment. Assessing stromal reactivity based on AP may therefore provide biologically relevant information beyond Gleason grading alone; for example, a distinct stromal signature within an intermediate-grade tumor—such as Pattern B—might indicate more aggressive behavior even if observed only focally. Although our study is descriptive, these mechanisms provide a plausible framework linking telocytic enrichment to the stromal remodeling and architectural disorganization observed in advanced prostate cancer.

Furthermore, tumor progression is significantly shaped by selective pressures from the microenvironment, rather than being determined exclusively by epithelial genomic instability. Classical evolutionary models of cancer progression suggest that adverse microenvironmental conditions, such as increased ECM density, altered stiffness, and limited nutrient diffusion, promote the development of aggressive tumor phenotypes. In contrast, more permissive environments support the coexistence of diverse cellular populations [32]. Therefore, tumor morphology and stromal architecture are emergent properties arising from biomechanical and biochemical constraints.

Several limitations should be acknowledged. Telocyte identification relied on immunophenotypic and morphological criteria rather than ultrastructural confirmation, and although the CD34^+^/Vimentin^+^ profile is widely accepted, it lacks absolute specificity. In addition, sampling was limited to sextant biopsies with one representative slide per case, and clinical endpoints such as biochemical recurrence, metastasis, or therapy response were not available. Future studies using whole-mount prostatectomy cohorts, integration with longitudinal clinical outcomes, and multimodal spatial approaches will be essential to validate the prognostic relevance of telocytic profiles and to determine whether stromal phenotyping can refine risk stratification beyond Grade Group alone.

## 4. Materials and Methods

### 4.1. Study Design

This study comprises a consecutive case series of 120 prostate biopsies prospectively accrued during routine diagnostic practice between 2017 and 2019 at Laboratório Analys Patologia (Belo Horizonte, MG, Brazil). All histopathological evaluations were independently performed by two experienced genitourinary pathologists, who reviewed all cases to confirm Gleason grading and architectural patterns according to current consensus criteria. Cases were included sequentially at the time of diagnosis, minimizing selection bias and reflecting real-world diagnostic practice. The cohort was evenly distributed across Gleason Grade Groups: GS 3 + 3 (Group 1, n = 30), GS 3 + 4 (Group 2, n = 30), GS 4 + 3 (Group 3, n = 30), and GS ≥ 8 (Group 4, n = 30). The mean patient age was 66 years (range, 43–89 years), and the median serum prostate-specific antigen (PSA) level was 7.5 ng/mL (interquartile range, 5.0–16.7 ng/mL; range, 1.4–2804 ng/mL).

Neoplastic architecture was categorized into four AP (A–D) using established criteria. Pattern A has well-formed glands with clear lumens. Pattern B features mostly rudimentary microacini or small, poorly formed glands, usually under 50 μm. Pattern C consists of complex epithelial sheets with multiple lumina, including all cribriform types. Pattern D is the most undifferentiated, made up of solid epithelial strands, nests, or isolated infiltrating cells. Additional details on cohort recruitment and clinicopathological characterization have been previously reported [17].

For each case, whole-slide digital images were acquired using the Pannoramic MIDI scanner (3D-HISTECH^®^—Budapest, Hungary). Stromal ROIs adjacent to neoplastic glands were randomly selected on hematoxylin–eosin–stained sections, following confirmation of tumor presence and architectural pattern. Selection was performed independently and blinded to clinical data and immunohistochemical results. This cohort has been characterized in detail in our previous publication (17).

The use of human tissue samples and patient data has been approved by the Ethics and Research Committee of the Federal University of Minas Gerais (COEP-UFMG; CAAE 09288112.0.0000.5149).

### 4.2. Sample Selection and Immunohistochemistry

For immunohistochemistry, one formalin-fixed and paraffin-embedded (FFPE) biopsy core per patient (n = 120) was selected from the six cores obtained per case through the standard sextant biopsy method. Selection prioritized the core containing the highest Gleason grade and largest tumor burden, in accordance with established criteria for reactive stroma assessment proposed by Ayala et al. [4], who recommend evaluating the most representative, highest-grade focus (index cancer) for reactive stroma quantification.

Serial sections (3–5 µm thick) were obtained from each selected FFPE sample and subjected to immunohistochemical staining for stromal markers. The following primary monoclonal antibodies were used: mouse anti-human α-smooth muscle actin (α-SMA, clone 1A4, Dako Denmark A/S, Glostrup, Denmark, 1:70), mouse anti-human CD34 (clone QBEnd-10, Dako Denmark A/S, Glostrup, Denmark, 1:100), mouse anti-human Desmin (clone D33, Dako Denmark A/S, Glostrup, Denmark, 1:100), and mouse anti-Vimentin (clone V9, Dako Denmark A/S, Glostrup, Denmark, 1:135). All staining procedures were performed on the Bond-max automated system (Leica Biosystems©—Wetzlar, HE, Germany) according to the manufacturer’s instructions. Immunoreactions were developed using DAB chromogen, and nuclei were counterstained with hematoxylin. Digitized whole-slide images were acquired using the Pannoramic MIDI scanner (3D-HISTECH^®^—Budapest, Hungary).

### 4.3. Image-Assisted Analysis of α-SMA, Desmin, Vimentin and CD34 Staining

Given that immunohistochemistry was performed on serial sections, ROIs initially defined on hematoxylin–eosin–stained slides were mapped onto the corresponding immunostained sections using digital slide alignment. This approach allowed spatial correspondence of stromal regions across different markers and minimized sampling bias due to tissue heterogeneity. Only regions with preserved morphology and clear correspondence across serial sections were included in the final analysis. For immunohistochemical quantification, at least two ROIs per case corresponding to stromal “hot spots” adjacent to tumor acini and were determined based on histological features indicative of reactive stroma [17]. After excluding regions lost during sectioning or lacking preserved tumor, 260 out of 327 ROIs were retained for the final immunohistochemistry analysis.

Quantification of immunohistochemical staining was performed using the IHC Profiler plugin in ImageJ (version 1.53; National Institutes of Health, Bethesda, MD, USA) [33]. Digital images of each ROI were acquired at 10x magnification on the Pannoramic MIDI scanner (3D-HISTECH^®^, Hungary). After color deconvolution to separate DAB and hematoxylin signals, pixel-wise intensity distributions were computed, and staining intensity was automatically categorized as 1 = negative, 2 = weak positive, 3 = moderate positive, and 4 = strong positive [33]. For each case, mean intensity scores across all ROIs were calculated and used for subsequent analyses.

To minimize interference from endothelial CD34-positive structures, vascular profiles were identified using the Vessel Analysis plugin and digitally excluded prior to IHC quantification. This ensured that analyses reflected stromal cell populations rather than vascular endothelium. The resulting images, with endothelial regions excluded, were subsequently analyzed using the IHC Profiler plugin, ensuring that quantification was restricted to the periacinar stromal compartment enriched in spindle-shaped stromal cells.

### 4.4. Multiplex Immunofluorescence Assay

Multiplex immunofluorescence was performed to confirm the presence of CD34^+^ cells outside blood vessels, thereby validating the identification of telocytes. Selected FFPE sections of non-neoplastic prostate tissue were subjected to antigen retrieval for 10 min at 110 °C in Tris-EDTA buffer (pH 9.0). Sections were then permeabilized in TBS containing 0.5% Triton X-100 for 15 min and blocked with 10% normal donkey serum. A primary antibody cocktail was applied for 2 h at room temperature, consisting of rat anti-CD34 (eBioscience/Invitrogen,—Carlsbad, CA, USA, #14-0341-82, 1:250), mouse anti-Vimentin (Proteintech, Rosemont, IL, USA, #60330-1-Ig, 1:2000), and goat anti-CD31 (R&D Systems—Minneapolis, MN, USA, #AF3628, 1:500). Secondary detection was performed for 1 h at room temperature with a mixture of species-specific Alexa Fluor–conjugated antibodies, diluted 1:400: rabbit anti-goat AF488 (Invitrogen, #A21222), donkey anti-rat AF555 (Jackson ImmunoResearch—West Grove, PA, USA, #712-565-153), and donkey anti-mouse AF647 (Jackson ImmunoResearch, #715-605-150). Fluorescent labeling was visualized using a Zeiss LSM 900 (Zeiss, Jena, Germany) confocal microscope with a 20× objective, acquiring 12 z-stacks at 0.8 μm intervals.

### 4.5. Statistical Analysis

Statistical analyses were performed in R version 4.5.2. Trends in the distribution of stromal phenotypes across ordered Gleason groups and AP were initially evaluated using the Cochran–Armitage chi-square test for trend. Monte Carlo simulations with 10,000 iterations were used to confirm *p*-value stability. These analyses were considered exploratory and descriptive in nature and were primarily used to support graphical summaries and hypothesis generation.

Ordinal immunohistochemistry scores (1–4) for α-SMA, Desmin, Vimentin, and CD34 were analyzed using cumulative link mixed models (CLMMs; logit link) fitted with the ordinal package (v2023.12.4.1; Ref. [34]). CLMMs are hierarchical ordinal regression models that allow the analysis of ordered outcomes while accounting for clustering through random effects. Each model included a patient-level random intercept to account for intra-patient variability, with Gleason group as the main predictor and age and serum PSA (both z-scored) included as covariates. Prior to model fitting, multicollinearity among predictors was assessed using variance inflation factors (VIF) from the car package (v3.1-3; Ref. [35]), which ranged from 1.06 to 1.20, indicating low collinearity. Model results were reported as odds ratios (OR) with 95% CI, and marginal predicted probabilities across Gleason groups were computed using the emmeans package (v2.0.0; Ref. [36]).

Bayesian multilevel logistic regression models were used to evaluate the association between stromal phenotypes (telocytic or myofibroblastic) and either APs or Gleason group. Acinar pattern was treated as an ordered factor (A–D) with linear, quadratic, and cubic polynomial terms. Gleason group (Group 1: GS 3 + 3; Group 2: GS 3 + 4; Group 3: GS 4 + 3; Group 4: GS ≥ 8) was modeled as an ordinal factor, allowing estimation of odds ratios for each group relative to the reference category (Group 1). All Bayesian models were adjusted for age and serum PSA (z-scored), included patient-level random intercepts, and were fitted using the brms package (v2.23.0; Ref. [37]) with a Bernoulli likelihood and logit link, employing four Markov chains with 4000 iterations each (1000 warm-up). Convergence was assessed using the potential scale reduction factor potential scale reduction factor (R-hat) and effective sample size. Importantly, priors were specified based on the observed distribution of binary events at both the ROI and patient levels, with effect sizes centered on log-odds differences suggested by empirical proportions. Groups with fewer observed events were assigned wider prior standard deviations to reflect greater uncertainty. Posterior draws were used to calculate odds ratios (ORs) and 95% credible intervals (CIs). Overall ORs for AP or Gleason groups correspond to the linear polynomial contrast summarizing the global trend across all levels. ORs for individual levels (Pattern B–D or Gleason Groups 2–4) were calculated from the full linear predictor, incorporating all polynomial contrast terms simultaneously, with the lowest category (Pattern A or Group 1) as reference. Predicted probabilities and pairwise comparisons were also derived from the posterior distribution.

## 5. Conclusions

Our histological and immunohistochemical analyses challenge the exclusively myofibroblastic identity of the RS in PCa, highlighting the pivotal role of telocytes in tissue organization, stromal compartmentalization, and collagen remodeling. These findings underscore the need for reconsidering the cellular landscape of the RS and open new paths for exploring telocyte-mediated signaling and potential stroma-targeted biomarkers. Specifically, the telocytic stromal phenotype (CD34^+^/Vimentin^+^) is significantly associated with advanced tumor morphology and AP B and D. By demonstrating that stromal characterization is critical for PCa risk stratification, we suggest that a stroma-based architectural analysis could complement the traditional Gleason scoring system by providing additional prognostic depth. Nevertheless, further large-scale longitudinal studies with patient follow-up and survival analysis are required to validate the clinical integration of these stromal signatures into routine practice.

## Figures and Tables

**Table 1:** Linear trends of stromal marker expression across Gleason Grade Groups assessed by ordinal logistic regression.

Marker	Estimate	Std. Error	OR	95% CI (OR)	*p*-Value
α-SMA	0.224	0.625	1.25	0.37–4.26	0.7210
Desmin	0.675	0.536	1.96	0.69–5.61	0.2080
CD34	2.091	0.539	8.09	2.81–23.30	0.0001
Vimentin	1.097	0.468	3.00	1.20–7.50	0.0190

Each stromal marker was modeled as an ordinal outcome predicted by Gleason Grade Groups using cumulative link mixed models (CLMM; logit link) with patient as a random intercept. Only the linear (L) component of each marker is shown; age and PSA covariates were excluded for simplicity. Complete model outputs are provided in Supplementary Table S1.

**Table 2:** Frequency of stromal phenotypes and Bayesian logistic regression estimates according to Gleason groups.

Variable	ROI Level		Patient Level		Bayesian Logistic Regression Model	
*Parameter*	*Present* *n (%)*	*Absent* *n (%)*	*Present* *n (%)*	*Absent* *n (%)*	*Predicted probability* *(95% CI)*	*Odds ratio* *(95% CI)*
**Telocytic phenotype**						
All Gleason groups	42 (16.2%)	218 (83.8%)	30 (25.0%)	90 (75.0%)	7.38% (2.16–15.76)	3.08 (1.31–6.25)
Group 1 (GS 3 + 3)	4 (6.6%)	57 (93.4%)	3 (10.0%)	27 (90.0%)	3.20% (0.81–7.52)	Reference
Group 2 (GS 3 + 4)	9 (13.8%)	56 (86.2%)	6 (20.0%)	24 (80.0%)	5.25% (1.41–11.95)	1.98 (0.55–5.10)
Group 3 (GS 4 + 3)	14 (20.9%)	53 (79.1%)	11 (36.7%)	19 (63.3%)	10.05% (2.98–20.90)	4.05 (1.17–10.25)
Group 4 (GS ≥ 8)	15 (22.4%)	52 (77.6%)	10 (33.3%)	20 (66.7%)	10.58% (3.30–21.83)	4.37 (1.26–11.23)
**Myofibroblastic phenotype**						
All Gleason groups	83 (31.9%)	177 (68.1%)	50 (41.7%)	70 (58.3%)	21.88% (10.87–35.41)	1.86 (0.92–3.36)
Group 1 (GS 3 + 3)	16 (26.2%)	45 (73.8%)	9 (30.0%)	21 (70.0%)	15.93% (7.20–27.88)	Reference
Group 2 (GS 3 + 4)	17 (26.2%)	48 (73.8%)	8 (26.7%)	22 (73.3%)	18.75% (9.41–30.49)	1.31 (0.62–2.48)
Group 3 (GS 4 + 3)	25 (37.3%)	42 (62.7%)	16 (53.3%)	14 (46.7%)	24.34% (12.47–38.31)	1.88 (0.77–3.82)
Group 4 (GS ≥ 8)	25 (37.3%)	42 (62.7%)	17 (56.7%)	13 (43.3%)	27.87% (14.03–44.13)	2.32 (0.86–5.10)

Frequencies are shown as number (%) at the ROI and patient levels. Predicted probabilities and odds ratios (ORs) were estimated from Bayesian multilevel logistic regression models (brms, logit link with patient as a random intercept; Age and PSA fixed at sample means). ORs for Gleason Groups 2–4 are shown relative to Group 1, calculated from the linear polynomial contrast coefficients of the Bayesian model. Observed trends across Gleason groups were generally supported by Cochran–Armitage tests (telocytic: ROI-level X^2^ = 6.96, *p* = 0.008; patient-level X^2^ = 6.01, *p* = 0.014; myofibroblastic: ROI-level X^2^ = 2.94, *p* = 0.087; patient-level X^2^ = 7.02, *p* = 0.008). Full model estimates are in Supplementary Table S2.

**Table 3:** Frequency of stromal phenotypes and Bayesian logistic regression estimates according to acinar pattern.

Variable	ROI Level		Patient Level		Bayesian Logistic Regression	
*Parameter*	*Present, n (%)*	*Absent, n (%)*	*Present, n (%)*	*Absent, n (%)*	*OR*	*95% CI*
**Telocytic phenotype**						
All patterns	57 (23.3%)	188 (76.7%)	40 (31.3%)	88 (68.8%)	5.55	1.78–13.97
Pattern A	7 (7.4%)	88 (92.6%)	6 (12.8%)	41 (87.2%)	Reference	Reference
Pattern B	33 (34.4%)	63 (65.6%)	23 (44.2%)	29 (55.8%)	15.39	3.77–45.10
Pattern C	2 (10.5%)	17 (89.5%)	2 (14.3%)	12 (85.7%)	2.13	0.25–8.15
Pattern D	15 (42.9%)	20 (57.1%)	9 (60.0%)	6 (40.0%)	29.20	5.04–104.33
**Myofibroblastic phenotype**						
All patterns	75 (30.6%)	170 (69.4%)	45 (35%)	83 (65%)	3.28	0.72–10.15
Pattern A	20 (21.1%)	75 (78.9%)	11 (23.4%)	36 (76.6%)	Reference	Reference
Pattern B	40 (41.7%)	56 (58.3%)	23 (44.2%)	29 (55.8%)	7.48	1.16–26.00
Pattern C	3 (15.8%)	16 (84.2%)	3 (21.4%)	11 (78.6%)	1.75	0.10–8.44
Pattern D	12 (34.3%)	23 (65.7%)	8 (53.3%)	7 (46.7%)	11.91	1.23–51.76

Frequencies are reported as number of observations with percentage in parentheses at the ROI and patient levels. Odds ratios (OR) and 95% CI were obtained from Bayesian multilevel logistic regression models (brms, logit link with patient as a random intercept; Age and PSA fixed at sample means). ORs for individual APs were calculated from the full linear predictor of the Bayesian logistic regression model, incorporating all polynomial contrast terms simultaneously and using Pattern A as reference. The full regression model including all covariates and estimates is presented in Supplementary Table S3.

## Data Availability

The datasets generated and analyzed during the current study are not publicly available because they are part of an ongoing project and are subject to ethical and privacy restrictions. Requests to access the data should be directed to the study administrator, Dr. Eduardo Paulino Junior (Hospital das Clínicas, Universidade Federal de Minas Gerais; email: edupatol@gmail.com).

## Supplementary Materials

The following supporting information can be downloaded at: https://www.mdpi.com/article/10.3390/ijms27031537/s1.

## Abbreviations

The following abbreviations are used in this manuscript:

PCa	Prostate cancer
RS	Reactive stroma
α-SMA	α-smooth muscle actin
AP	Acinar patterns
ROIs	Regions of interest
CLMM	Cumulative link mixed models
PCA	Principal component analysis
ICLCs	Interstitial Cajal-like cells
CAFs	Cancer-associated fibroblasts
myCAFs	Myofibroblast-like CAFs
OR	Odds ratio
CI	Credible interval
IQR	Interquartile range
