# Supplementary material for: Immunohistochemical Evidence of Telocytic Stroma Associated with Tumor Grade and Acinar Heterogeneity in Prostate Cancer
Source: Int J Mol Sci. 2026 Feb 4;27(3):1537. doi: 10.3390/ijms27031537 (PMC12897662; doi:10.3390/ijms27031537)
Supplement: Supplementary file 1 [file ijms-27-01537-s001.zip › ijms-3905080-supplementary.pdf]

Supplementary Table S1: Ordinal mixed-effects regression analysis of stromal marker expression across Gleason grade groups in prostate cancer

Marker	Component	Estimate	Std. Error	Z value	OR	95% CI (OR)	p-value
SMA	Gleason_group.L	0.224	0.625	0.358	1.25	0.37–4.26	0.721
SMA	Gleason_group.Q	0.082	0.613	0.134	1.09	0.33–3.61	0.893
SMA	Gleason_group.C	-0.642	0.618	-1.039	0.53	0.16–1.77	0.299
SMA	Age(z)	-0.342	0.314	-1.09	0.71	0.38–1.31	0.276
SMA	PSA(z)	-0.426	0.291	-1.461	0.65	0.37–1.16	0.144
Desmin	Gleason_group.L	0.675	0.536	1.259	1.96	0.69–5.61	0.208
Desmin	Gleason_group.Q	0.415	0.525	0.79	1.51	0.54–4.24	0.43
Desmin	Gleason_group.C	-0.523	0.521	-1.003	0.59	0.21–1.65	0.316
Desmin	Age(z)	0.043	0.264	0.162	1.04	0.62–1.75	0.872
Desmin	PSA(z)	0.062	0.238	0.262	1.06	0.67–1.70	0.793
CD34	Gleason_group.L	2.091	0.539	3.878	8.09	2.81–23.27	0.0001
CD34	Gleason_group.Q	-1.023	0.473	-2.162	0.36	0.14–0.91	0.031
CD34	Gleason_group.C	0.269	0.44	0.611	1.31	0.55–3.10	0.541
CD34	Age(z)	0.078	0.233	0.337	1.08	0.69–1.71	0.736
CD34	PSA(z)	0.064	0.199	0.322	1.07	0.72–1.58	0.748
Vimentin	Gleason_group.L	1.097	0.468	2.345	3	1.20–7.50	0.019
Vimentin	Gleason_group.Q	0.111	0.448	0.248	1.12	0.46–2.69	0.804
Vimentin	Gleason_group.C	-0.046	0.446	-0.104	0.95	0.40–2.29	0.917
Vimentin	Age(z)	-0.117	0.228	-0.512	0.89	0.57–1.39	0.608
Vimentin	PSA(z)	-0.116	0.214	-0.539	0.89	0.59–1.36	0.59

Cumulative link mixed models (CLMM; logit link) were fitted separately for each stromal marker (SMA, desmin, CD34, and vimentin). Marker expression was modeled as an ordinal outcome (scores 1–4: negative, weak, moderate, high), and Gleason grade groups were included as an ordered predictor (Group 1: GS 3+3; Group 2: GS 3+4; Group 3: GS 4+3; Group 4: GS ≥ 8). Linear (L), quadratic (Q), and cubic (C) terms represent orthogonal polynomial contrasts across Gleason groups. Models were adjusted for z-score–scaled age and PSA levels and included patient as a random intercept to account for repeated measurements.

Supplementary Table S2: Bayesian multilevel logistic regression models for stromal phenotypes according to Gleason group

Term	Estimate	Est. Error	OR	95% CI	Post. Prob.
**Telocytic phenotype**					
Intercept	−2.78	0.45	0.07	0.02–0.14	0.000
Gleason group – Linear (L)	1.05	0.40	3.09	1.31–6.26	0.996
Gleason group – Quadratic (Q)	−0.23	0.38	0.85	0.37–1.70	0.268
Gleason group – Cubic (C)	−0.19	0.38	0.89	0.39–1.78	0.305
Age_z	−0.21	0.29	0.85	0.45–1.45	0.233
PSA_z	−0.54	0.52	0.66	0.17–1.34	0.131
**Myofibroblastic phenotype**					
Intercept	−1.35	0.31	0.27	0.13–0.46	0.000
Gleason group – Linear (L)	0.57	0.33	1.86	0.92–3.36	0.960
Gleason group – Quadratic (Q)	−0.01	0.27	1.02	0.58–1.65	0.478
Gleason group – Cubic (C)	−0.06	0.19	0.96	0.65–1.36	0.370
Age_z	−0.07	0.26	0.97	0.56–1.57	0.399
PSA_z	−0.38	0.32	0.72	0.35–1.24	0.113

Bayesian multilevel logistic regression models were used to assess associations between stromal phenotypes (telocytic or myofibroblastic) and Gleason groups (Group 1: GS 3+3; Group 2: GS 3+4; Group 3: GS 4+3; Group 4: GS ≥ 8) treated as ordinal factor. Models included linear, quadratic, and cubic polynomial terms for Gleason group, adjusted for age and PSA (z-scored), and incorporated patient-level random intercepts. Posterior draws were used to estimate odds ratios, 95% credible intervals (CI), and posterior probabilities.

Supplementary Table S3: Bayesian multilevel logistic regression models for stromal phenotypes according to acinar pattern

Term	Estimate	Est. Error	OR	95% CI	Post. Prob.
**Telocytic phenotype**					
Intercept	−2.00	0.39	0.15	0.06–0.27	0.000
Acinar pattern – Linear (L)	1.57	0.53	5.55	1.78–13.97	0.999
Acinar pattern – Quadratic (Q)	0.09	0.54	1.27	0.38–3.28	0.561
Acinar pattern – Cubic (C)	2.13	0.66	10.6	2.50–33.04	0.999
Age_z	−0.18	0.28	0.87	0.48–1.48	0.259
PSA_z	−0.12	0.29	0.93	0.47–1.55	0.349
**Myofibroblastic phenotype**					
Intercept	−1.95	0.49	0.16	0.05–0.35	0.000
Acinar pattern – Linear (L)	0.96	0.66	3.28	0.72–10.15	0.929
Acinar pattern – Quadratic (Q)	0.19	0.71	1.56	0.30–4.94	0.600
Acinar pattern – Cubic (C)	1.61	0.78	6.86	1.11–24.34	0.981
Age_z	0.09	0.42	1.20	0.48–2.55	0.583
PSA_z	−0.77	0.59	0.55	0.13–1.34	0.081

Bayesian multilevel logistic regression models were used to assess associations between stromal phenotypes (telocytic or myofibroblastic) and acinar pattern (A–D). Models included linear, quadratic, and cubic polynomial terms for acinar pattern, adjusted for age and PSA (z-scored), and incorporated patient-level random intercepts. Posterior draws were used to estimate odds ratios, 95% credible intervals (CI), and posterior probabilities.
